# Nano-emulsification of oriental lacquer sap by ultrasonic wave propagation: Improvement of thin-film characteristics as a natural resin

**DOI:** 10.1016/j.ultsonch.2021.105545

**Published:** 2021-03-29

**Authors:** Hyo-Jun Oh, Jun Ho Hwang, Minju Park, Soo Jin Kim, Jihoo Lee, Ho Sun Lim, Sang-Soo Lee, Jung Ah Lim, Eunji Lee

**Affiliations:** a School of Materials Science and Engineering, Gwangju Institute of Science and Technology (GIST), Gwangju 61005, Republic of Korea; b Photo-Electronic Hybrids Research Center, Korea Institute of Science and Technology (KIST), Seoul 02792, Republic of Korea; c Department of Chemical and Biological Engineering, Sookmyung Women’s University, Seoul 04310, Republic of Korea

**Keywords:** Lacquer sap, Ultrasonic waves, Nano-emulsification, Rapid polymerization, High transparency, Strong adhesion

## Abstract

Lacquer sap has received much attention as a traditional natural resin because it is a renewable and eco-friendly biopolymer resource unlike artificial coating materials. However, strict drying conditions and long drying times of lacquer sap should be modified to expand its applications. This study presents the first attempt to investigate the effect of different amplitudes of ultrasonic waves on the lacquer sap composed of water-in-oil (W/O) emulsion droplets and the mechanical properties of the resultant film by solvent evaporation. Acoustically induced cavitation via batch ultrasonication facilitates the generation of submicron-sized W/O emulsion. The drying time of sonicated lacquer sap was noticeably shortened as the amplitude of acoustic power increased. Interestingly, the transparency of the film cast from lacquer sap consisting of the smallest emulsion droplets increased significantly, weakening the degree of colour change from caramel-like yellow to dark brown as polymerisation progressed. These are attributed to the effective and frequent contact of laccase enzyme with urushiol at the increased interfacial area of nano-emulsified W/O droplets pulverised by ultrasonic waves. The quinone radical-generation in the interface layer and its transfer to the urushiol oil phase through water-insoluble glycoprotein emulsifier are greatly promoted, resulting in highly cross-linked, dense three-dimensional polymer networks, which also increased the lacquer film hardness after drying. As the emulsion droplet size decreased, the mutual interaction between the catechol moiety of urushiol and the substrates increased, resulting in improved adhesion. The nano-emulsification of the lacquer sap by ultrasonic waves can be used in a simple, effective, and eco-friendly way to shorten the drying time and improve the film characteristics of natural resins. This approach could pave the way for its wide range of applications in industrial fields, taking into account green and sustainable chemistry.

## Highlights

• Raw lacquer sap emulsion droplets shrink as ultrasonication intensity increased.
• Nano-emulsified lacquer reduces drying time with promoted urushiol polymerisation.
• Increased urushiol–laccase contact for enzymatic oxidation reduces discoloration.
• Reduced light scattering by nano-emulsion aids high transmittance of lacquer film.
• Small droplets aid strong urushiol networks and catechol substrate interaction.

## Graphical abstract

**Figure:**
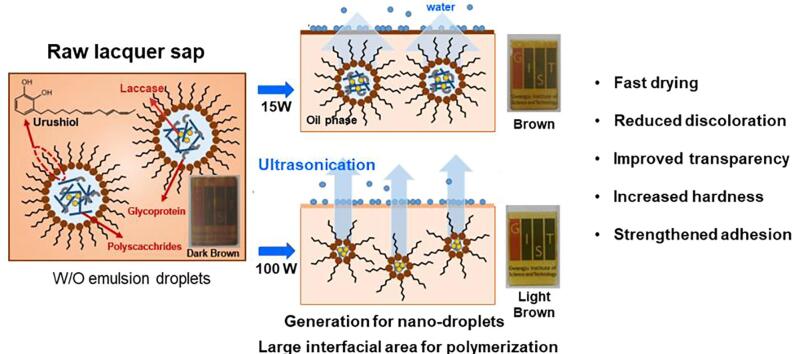

## 1 Introduction

The enzyme-catalysed polymerisation of oriental lacquer sap collected from lacquer trees in Asia allows it to be used as an energy-saving and environmentally friendly coating material with antibacterial, antiseptic, waterproof, and chemically resistant actions [1], [2], [3], [4], [5], [6], [7]. The lacquer thin films ensure excellent physicochemical properties such as high thermal stability, superior durability, anti-corrosiveness, good adhesion, and high gloss [8], [9], [10]. However, the drying process, which requires severe and strict conditions (approximately 70–90% relative humidity (RH) at 20–30 °C), has been issued a critical factor that hinders practical application in industrial fields, even though it is a biodegradable, renewable resource. The long drying time of lacquer sap is caused by stable diene bonds of urushiol, which is a main lacquer constituent [2], [11], [12]. Moreover, the resultant film is severely discoloured from caramel-like yellow to turbid deep brown as the polymerisation process is completed [13]. Inevitably, the discolouration, gloss reduction, and lacquer film deterioration can be caused by poor environmental conditions such as exposure to both fluorescence and visible light [14]. These problems associated with the lacquer sap make it difficult to apply practically as coating resins despite its excellent functions and eco-friendliness.

**Fig. 1:**
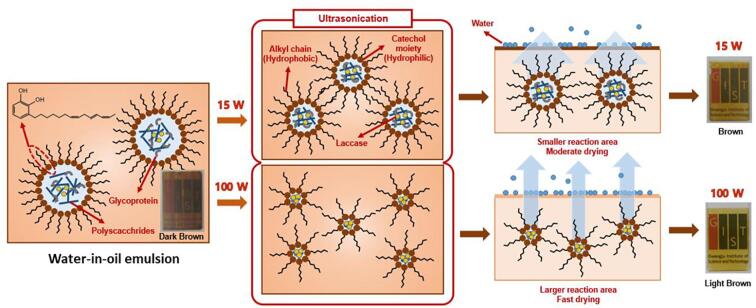
Schematic representation showing the effect of nano-emulsification of lacquer sap solution by ultrasonic wave on the film property.

The main component is the urushiol (60–65% in the sap), which consists of structurally similar 1,2,3-tri-*O*- or 1,3-di-*O*-substituted catechol derivatives with a long alkyl side chain with unsaturated double bonds (Fig. 1). The lacquer sap consists of water-in-oil (W/O) emulsion droplets [15], [16], [17]. Urushiol, urushiol-laccase, -stellacyanin complexes, and water-insoluble glycoprotein exist in the interlayer between the two phases. The glycoprotein and polysaccharide-containing water act as an emulsifier and dispersed phase, respectively. The curing process has generally been interpreted as a two-step process following solvent evaporation without taking into account the emulsion form of lacquer sap [12]. The laccase-catalysed oxidative coupling of urushiol with a phenol moiety occurs to form oligomers at room temperature and high humidity. Subsequently, the double bonds in the side chains contribute to a highly cross-linked structure by aerobic oxidation. Meanwhile, the polysaccharide inhibits rapid evaporation of water, which in turn contributes to the maintenance of laccase enzyme activity. However, based on the emulsion form of lacquer sap, Yang et al. explained that the radicals generated by the laccase-catalysed oxidation of urushiol at the interface layer can be transferred to the urushiol in the oil phase via water-insoluble glycoprotein, which initiates the polymerisation of urushiol in the oil phase [16]. At the interlayer, stellacyanin inhibits certain radicals, suppressing urushiol polymerisation at the W/O emulsion interlayer of the lacquer sap. The reduced laccase is oxidised by oxygen, which easily penetrates the water phase to induce continuous polymerisation. In this context, the drying time, drying conditions, and mechanical properties of the lacquer film should be improved based on a better understanding of lacquer sap components consisting of W/O emulsion droplets and curing processes from the perspective of soft matter materials chemistry.

To address the above issues, many efforts have been made to induce the rapid polymerisation of lacquer sap by controlling the composition of components or using additives [11], [18], [19]. Lu et al. reported on repeated *kurome*-lacquers with shortening drying time under ambient conditions [18]. The raw lacquer drying time was reduced by promoting enzyme oxidation through the *kurome* process, which reduces the raw lacquer water content by agitating and heating it to 40 °C. The repeated *kurome* process involves the prepolymerisation of urushiol, which produces a large number of polymers in lacquer. They also demonstrated that the addition of organic functional silane to lacquer sap leads to a fast alcoholysis with urushiol and promotes polymerisation, resulting in the improvement of the drying process-based laccase activity that significantly affects enzyme polymerisation [11]. In addition, Yang et al. explained that pure oxygen exposure to lacquer sap accelerates urushiol polymerisation by catalysing the oxygen reduction at a trinuclear copper laccase cluster containing four copper atoms [19]. However, such methods provide a time-consuming process or impair the nature of environmentally friendly materials. For this reason, many researchers have attempted to reduce the drying time by simply improving the processing without changing the lacquer components [10], [20], [21]. The prepolymerisation of urushiol was induced by UV irradiation and high temperature. However, lacquer film properties, such as gloss and durability, are severely damaged and may cause film deterioration of the lacquer surface, such as discolouration, fading, cracking, and lifting.

Herein, we report on the facile process of achieving fast drying time by applying ultrasonic waves considering the W/O emulsion droplet formulation of lacquer sap. The nano-emulsification of lacquer sap controlled by the amplitude of acoustic power inducing the cavitations has dramatically reduced the drying time and greatly improved transmittance, colour, hardness, gloss, and adhesion of lacquer film, thereby promoting the large-scale application of lacquer sap as natural-derived industrial coating and paint materials.

## 2 Experimental methods

### 2.1 Materials

A lacquer sap was purchased from Fuji Lacquer Craft Co., Ltd., Fukui, Japan. Turpentine oil, with its main components of *α*-pinene (46.6%) and *β*-pinene (1.15%), was purchased from Pine Nghe, A Joint Stock Company in Vinh, Vietnam. Glass slides (super grade) and red pine woods were purchased as substrates for lacquer coating from Citotest Labware Manufacturing Co., Ltd. in Jiangsu, P. R. China, and Hwainart Company in Seoul, Republic of Korea. A human hair brush for brush coating was purchased from Fuji Lacquer Craft Co., Ltd. in Japan.

### 2.2 Methods

Branson 2510 Ultrasonic Cleaner (2510E-DTH, Output Power HF: 100 W, Frequency: 42 kHz, Branson Ultrasonics Co., Ltd., USA), and GT Sonic Ultrasonic Cleaner (GT-X1, Output Power HF: 15 W, Frequency: 40 kHz, Guangdong GT Ultrasonic Co., Ltd., P. R. China) were used to apply the ultrasonic waves to the lacquer sap solution. The W/O emulsion of the lacquer sap solution was observed using an optical microscope (Eclipse LV100, Nikon Co., Ltd., Japan) equipped with a digital camera (DS-Ri1, Co., Ltd., Japan). The Image J program was used to measure the size distribution of the emulsion droplets. The emulsion droplet diameter was further evaluated using a dynamic light scattering spectrometer (DLS, ELSZ-2000, Otsuka Electronics Co., Ltd., Japan). A digital incubator (MX-20, Autoelex Co., Ltd., Republic of Korea) capable of constantly controlling temperature and humidity was used to measure the drying time of the lacquer sap to film while maintaining a temperature of 27.5 °C and 80% RH. The drying time was measured using touch-free drying (TD) and hardened drying (HD) methods according to the International Organisation and Standardisation 9117 (ISO 9117). The lacquer sap solution polymerisation was monitored using a Fourier transform infrared spectrophotometer (FT-IR-8400S, Shimadzu Co., Ltd., Japan). The moisture content of the raw lacquer sap was determined by moisture analysis balance (i-Thermo 163 M, Bel Engineering, Italy). UV–Vis transmittance was measured with a double beam spectrophotometer (UH5300, Hitachi High-Tech Co., Ltd., Japan). The dried lacquer film thickness was measured using a thin-film mechanical profilometer (Alpha Step-IQ, KLA Tencor Instruments, USA). The film hardness was determined using a pencil hardness tester (BGD 506, Biuged Laboratory Instruments Co., Ltd., P. R. China) following ASTM D3363 standard. The peel adhesion strength was determined according to the national standard ASTM D3359 tape test using a crosscutter (SS-YCC1, Yoshimitsu, Japan). The gloss was tested with a 20°–60°–85° specular glossmeter (Novo-Gloss Trio, Rhopoint Instruments, UK). The cross-sectional film view was acquired using a field emission-scanning electron microscope (FE-SEM, JSM-7500F, JEOL Co., Ltd., Japan).

## 3 Results and discussion

### 3.1 Nano-emulsification of lacquer sap solution by sonication

**Fig. 2:**
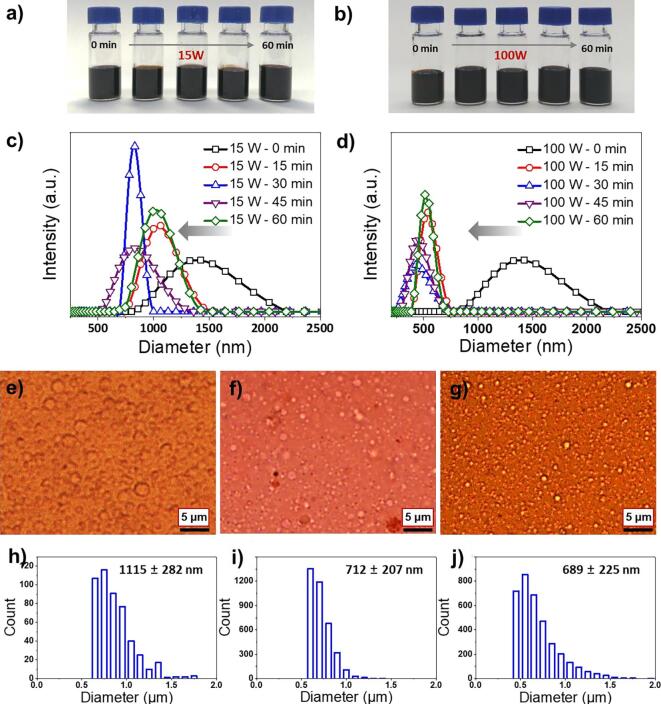
Micrographs showing the lacquer sap solution under different sonication power output. of a) 15 W and b) 100 W as a function of time (15 min interval). Hydrodynamic radius of lacquer sap solution under different ultrasonic waves of c) 15 W and d) 100 W. Optical micrographs of ultrasonicated lacquer sap solutions treated e, f) with the power of 15 W for e) 15 and f) 30 min, and (g) 100 W for 30 min. h–j) The size distribution of emulsion droplets observed in each photograph of e), f), and g).

Fig. 1 shows the effect of sonication power on the W/O emulsion droplet size that comprises the lacquer sap solution, drying process, and the resultant film properties. Initially, the lacquer sap solution was prepared with turpentine oil extracted from pine trees (a 1:1 (v/v) solution of raw lacquer sap and turpentine oil) because of the high viscosity of the raw lacquer sap with 2927 cps at 26 °C. It is difficult to induce cavitation in raw lacquer sap-emulsion droplets by sonication [22]. It is well known that numerous bubbles are formed when sonication is applied because the ultrasonic wave has a short wavelength and high energy [23]. When the ultrasonic wave pressure exceeds the bubble surface tension, the bubbles implode, causing local high temperature and pressure, which is called the cavitation phenomenon. This implosion generates a shock wave, creating a jet stream of the surrounding solution, pressurising the dispersed droplets, and reducing their size. To confirm the presence of cavitation effect on the lacquer sap solution, ultrasonic waves of 15 W ultrasonic power at a fixed frequency of 40 kHz (Fig. 2a) were used. Droplet sizes were measured by dynamic light scattering (DLS) as a function of processing time from 0 to 60 min. The dynamic droplet diameter decreased with increasing sonication time, as shown in Fig. 2c, and the size distribution was more homogeneous [24], [25]. The size of the droplets dispersed in the oil phase was also measured from optical micrographs using the Image J program (Fig. 2e–j). At processing times of 15 and 30 min, the diameter gradually decreased from 1115 ± 282 to 712 ± 207 nm (Fig. 2h, i), respectively, which is consistent with the DLS data.

The lacquer sap solution was also sonicated at a different amplitude of 100 W ultrasonic power (at a fixed frequency of 40 kHz, Fig. 2b) [26]. The processing volume was the same as that at 15 W. DLS data showed the formation of smaller W/O droplets when treated with 100 W power intensity compared to the lacquer sap solution treated with ultrasonic waves of 15 W over a given period time (Fig. 2d). These results were also identified in the size distribution graph measured quantitatively in a micrograph. For sonication for 30 min, a droplet size of 689 ± 225 nm was measured (Fig. 2j). The lacquer sap solution treated by ultrasonication showed smaller and uniform-sized W/O droplets compared to the bare lacquer sap solution. Interestingly, when 15 W ultrasonic waves were applied to the lacquer sap solution, the droplet size was no longer reduced and slightly increased after 45 min. The same phenomenon was observed after 30 min of treatment with ultrasonic waves of 100 W. This is due to partial re-aggregation of the droplets, resulting in an increase in the droplet size [24], [27]. As the W/O droplet size decreased by sonication, the polymerisation of urushiol occurred actively in the lacquer sap solution, resulting in the coalescence of droplets due to the insufficient amount of urushiol that stabilizes the emulsion interface. It is interpreted that this leads to inhomogeneity of lacquer sap solution and consequently has a poor effect on improving thin film properties. It is indicated that the proper treatment time according to the sonication power should be considered in the nano-emulsification of lacquer sap. Detailed experimental results related to film properties will be covered in the next chapter.

### 3.2 Characteristics of thin films with nano-emulsified lacquer sap solution

#### 3.2.1 Drying time and colour

**Fig. 3:**
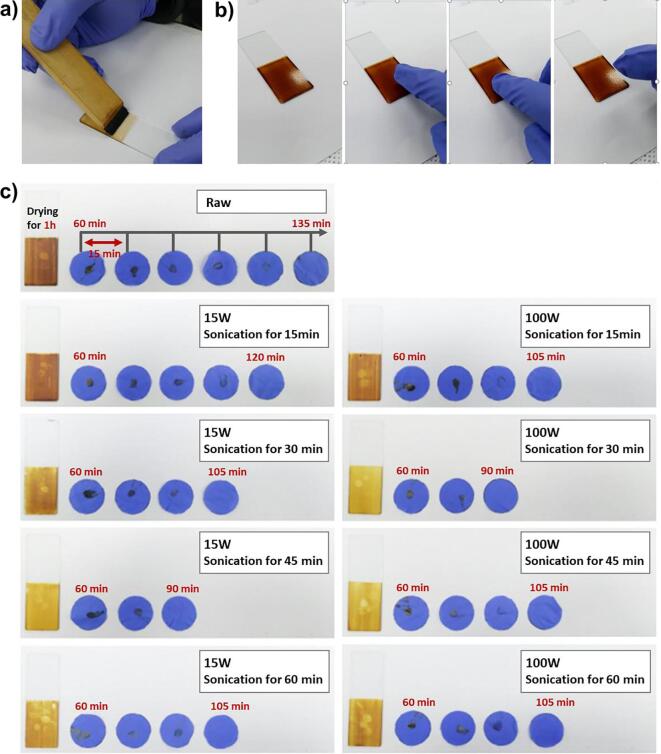
Drying time of nano-emulsified lacquer sap solution (27.5 °C, 80% RH). a) Painting of the lacquer sap solution on the glass. b) Touch-free drying (TD) method for drying time evaluation. c) Representing the drying time according to the ultrasonic power and processing time. The degree of drying was measured every 15 min after an initial 1 h.

**Fig. 4:**
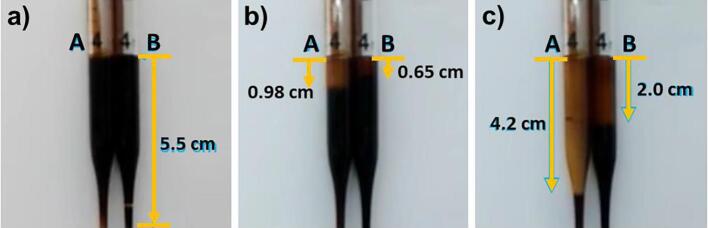
Viscosity measurements of (A) raw and (B) ultrasonicated (100 W for 30 min) lacquer sap solutions: a) ready to drop, b) after 1 s, and c) 2 s.

The nano-emulsification of lacquer sap solution provides a smaller diameter of W/O emulsion droplets, securing a greater contact area between urushiol and laccase [1]. We hypothesised that enzyme-catalysed polymerisation can occur more easily and induce rapid drying time. In this context, we investigated the drying time of the lacquer films fabricated with different amplitudes (15 and 100 W) and processing times (15, 30, 45, and 60 min) of sonication at 27.5 °C and 80% RH (Fig. 3). The lacquer sap solution used in this study was completely dried within 135 min as measured by the TD method (Fig. 3b). This was confirmed every 15 min. The drying times of the lacquer films ultrasonicated by 15 W power intensity for 15, 30, 45, and 60 min were shown to be 120, 105, 90, and 105 min, respectively (Fig. 3c). The shortest drying time was observed when the lacquer sap solution was processed for 45 min, which is equal to the time observed for the smallest emulsion droplet formation. In addition, the drying times of the film of a lacquer sap solution with 100 W ultrasonic waves for 15, 30, 45, and 60 min was confirmed to be 105, 90, 105, and 105 min, respectively (Fig. 3c). For the 30 min ultrasonication treatment, where the smallest emulsion droplets were observed, the drying time was found to be the shortest. These results clearly indicate that the W/O emulsion droplet size greatly affects the drying time. The urushiol of lacquer sap solution is cured by a laccase-catalysed reaction and an aerobic oxidation reaction, so that the water content of W/O emulsion droplets containing laccase enzymes also plays an important role in the polymerisation of urushiol, as mentioned previously [1], [12]. Table 1 shows the moisture content of the lacquer sap solution by comparing the weight before and after drying. The lacquer sap solution without ultrasonication treatment shows a moisture content of 20.0%, but the lacquer sap solution treated by sonication of 15 W and 100 W represent 12.5% and 10.4%, respectively. This means that the nano-emulsion formation of the lacquer sap solution by ultrasonication allows a smaller amount of water within the W/O emulsion droplets and consequently gives rise to the fast drying process. As the ultrasonic wave intensity increases, the number of droplets in contact with the atmosphere increases significantly, and the moisture reduction rate increases due to the cavitation effect. This leads to rapid urushiol curing at the droplet W/O interface while reacting with oxygen in the atmosphere [19], [28]. Interestingly, the films formed from raw lacquer sap were discoloured to deep brown, while films formed from nano-emulsified lacquer sap solution showed a homogeneous yellow colour (Fig. 1, Fig. 3c). In addition, when we pre-treated the lacquer sap solution for 30 min with sonication of 100 W amplitude and confirmed the solution viscosity using a glass capillary (Fig. 4), the solution was found to be more viscous after sonication. This can be explained by the fact that the W/O emulsion droplets comprising the lacquer sap solution have relatively increased contact with oxygen during sonication, resulting in facilitation of radical transfer-driven prepolymerisation of urushiol in the solution. The solution drying time was reduced due to the oxidation-driven prepolymerisation of urushiol.

**Table 1:** Moisture contents of raw and ultrasonicated lacquer sap solutions.

Samples	Before drying (g)	After drying (g)	Moisture (%)
Lacquer sap solution^a^	6.0	4.8	20.0
15 W treatment	5.4	4.8	12.5
100 W treatment	5.3	4.8	10.4

a The solution was prepared with raw lacquer sap and turpentine oil in a 1:1 (v/v) ratio.

#### 3.2.2 Polymerisation of lacquer films

**Fig. 5:**
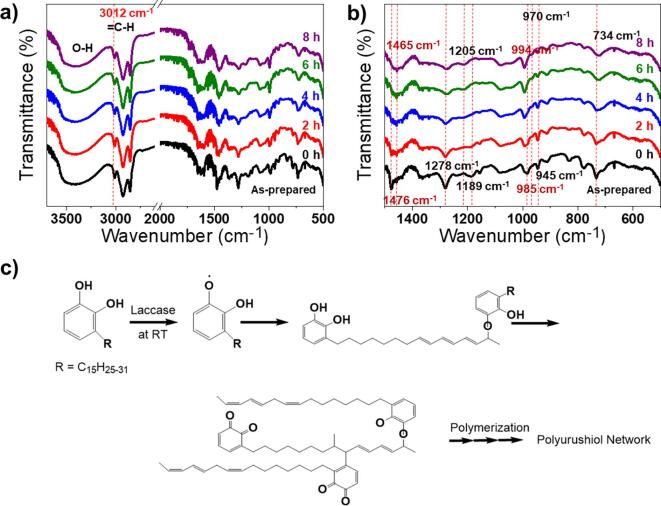
FT-IR spectra of ultrasonicated (100 W for 30 min) lacquer dried in 0–8 h: a) full spectrum, b) the partial spectrum of a) ranging from 500 to 1500 cm^−1^, and c) cross-linking process of lacquer by urushiol polymerisation.

To further investigate the polymerisation of urushiol-based lacquer sap, FT-IR spectroscopy was performed with cured lacquer films (Fig. 5). The sonicated lacquer sap solution was coated on a ZnSe window and dried at 27.5 °C and 80% RH. Notably, the quinone-radicals generated from laccase-catalysed urushiol transfers to form oligomers, and the cross-links of long aliphatic unsaturated chains by auto-oxidation with oxygen leads to lacquer polymerisation [12], [18]. As a result, the peak intensities of 1278 cm^−1^ and 1189 cm^−1^ due to *γ*O–H and C–O vibration of the C–O–H group gradually decreased, while a broad peak at 1205 cm^−1^ appeared instead of a peak at 1189 cm^−1^ with an increase in polymerisation, indicating that the O–H of the phenyl ring was involved in the polymerisation [3], [19], [21]. Indeed, the decrease in the peak at 734 cm^−1^ indicates an additional substitution on the phenyl ring. The peaks at 985 cm^−1^ and 945 cm^−1^ ascribed to the conjugated dienes noticeably decreased. The peak at 985 cm^−1^ corresponding to the non-conjugated double bond absorption triene shifted to 994 cm^−1^ within 4 h due to the formation of the conjugated triene double bond with an increase in drying time [11], [18]. The ether combination of urushiol at 1465 cm^−1^ appeared, suggesting that conversion of the O–H of urushiol into the quinone-radical was successfully generated and side chain oxidisation for the chain bridge occurred. This confirmed that the enzyme-catalysed polymerisation reaction of nano-emulsified lacquer sap successfully occurred within 4 h.

#### 3.2.3 Transmittance and gloss

**Fig. 6:**
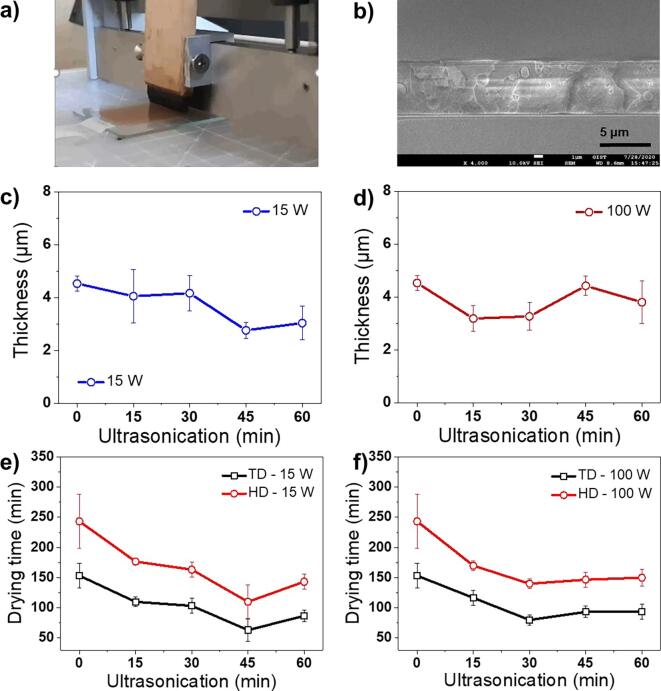
The thickness and drying time of films as cast from lacquer sap solution with brush coater. a) Micrograph for brush coating of lacquer sap solution and b) SEM image for the cross-sectional view of lacquer film. The thickness of lacquer films prepared with ultrasonicated lacquer sap solution: c) 15 W and d) 100 W treatments. e, f) Graphs for drying time of ultrasonicated lacquer sap solution with different power intensity of e) 15 W and f) 100 W (measured by TD and HD methods).

It is well known that the emulsion size is closely related to the resultant film thickness [29]. The brush coater was applied to reduce the lacquer film thickness error according to the applied coating method, and the thickness was measured using an Alpha-Step (Fig. 6a). The cross-sectional view of the lacquer film obtained by scanning electron microscopy showed that the film produced using a brush coater had a regular thickness (Fig. 6b). The raw lacquer film thickness was estimated as 4.53 μm. The thickness of the ultrasonicated lacquer films treated for 15, 30, 45, and 60 min with 15 W and 100 W amplitudes was measured as 4.05, 4.16, 2.76, 3.03 μm and 2.96, 2.88, 4.42, and 3.80 μm, respectively (Fig. 6c, d). The drying time of the ultrasonicated lacquer sap solution treated for 15, 30, 45, and 60 min with 15 W and 100 W amplitudes was measured as 110, 103, 63, and 87 min and 116, 80, 93, and 93 min, respectively (Fig. 6e, f). The drying times measured in TD and HD are shown in Fig. 6 and Table 2. Overall, the film thickness significantly reduced as the emulsion droplet size decreased, shortening the drying time. This is evident from the fact that the thickness and drying time of the lacquer film increased slightly (Fig. 6e, f) when the emulsion droplet size increased slightly in a given time for ultrasonication treatment (45 min for 15 W; 30 min for 100 W, Fig. 2c, d).

**Table 2:** Characteristics of lacquer thin film prepared with raw and ultrasonicated lacquer sap solutions.

Samples	Ultrasonication (min)	TD (min)	Thickness (μm)	Transmittance (% @600 nm)	Glossy (% @60°)	Pencil hardness
Lacquer sap solution	0	150	4.53	53.2	56.0	H
15 W treatment	15	110	4.05	64.5	60.0	H
	30	103	4.16	68.4	63.0	H
	45	63	2.76	74.2	55.0	2H
	60	87	3.03	71.6	60.1	H
100 W treatment	15	116	2.96	70.4	52.6	H
	30	80	2.88	70.8	56.3	2H
	45	93	4.42	67.5	61.6	H
	60	93	3.80	64.8	63.2	HB

**Fig. 7:**
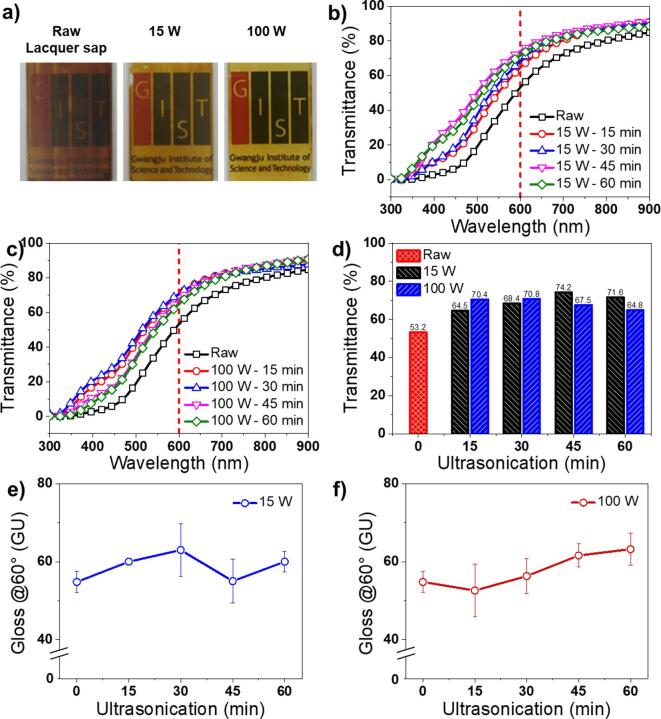
Change in a) colour, b-d) transmittance, and e, f) glossy of lacquer films, prepared using raw and ultrasonicated lacquer sap solutions.

The lacquer film transmittance was measured with samples coated on the slide glass using a brush coater (Fig. 7a). The lacquer film prepared with raw lacquer sap solution showed a transmittance of 53.2% at a wavelength of 600 nm (Fig. 7b–d). The lacquer film transmittance prepared with ultrasonicated solution with 15 W power intensity was measured at the least 64.5% and up to 74.2% (Fig. 7b, d). The lacquer film transmittance prepared by ultrasonication at 100 W intensity was measured at the least 64.8% and up to 70.8% (Fig. 7c, d). The lacquer film transmittance was improved when using the ultrasonicated lacquer sap solution due to the reduction in scattering originating from the generation of smaller emulsion droplets [30]. In addition, the raw lacquer film gloss was 56.0, but that of the nano-emulsified lacquer-films prepared by ultrasonication with 15 W and 100 W amplitudes were improved up to 60.1 and 63.2, respectively (Fig. 7e, f). It is noted that the numerical value is the ratio of the reflected light on the lacquer film surface to the standard substrate. This can be explained by the formation of smaller W/O emulsion droplets in the lacquer sap solution when treated with ultrasonic waves. The data on drying time, thickness, transmittance, and gloss are summarised in Table 2.

#### 3.2.4 Hardness and adhesion

**Fig. 8:**
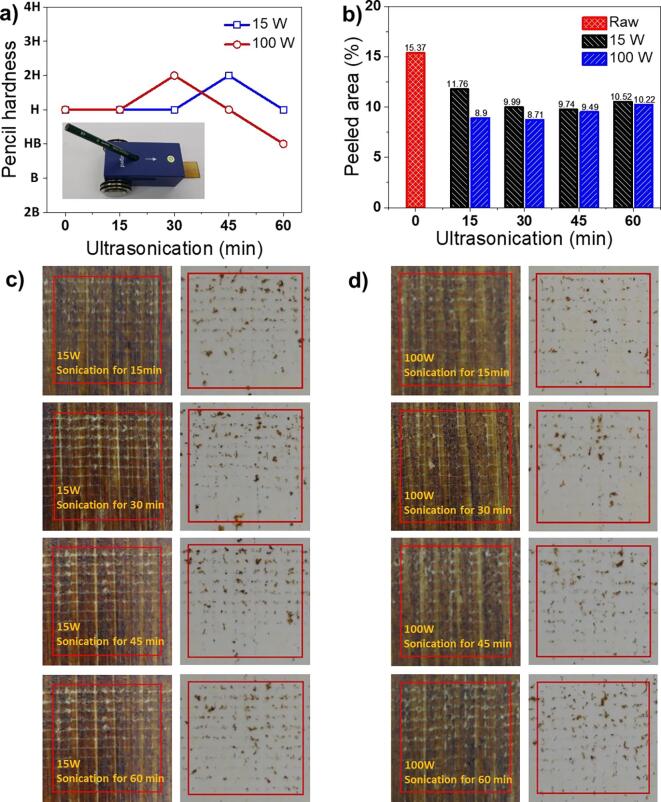
a) Pencil hardness and b–d) adhesion measurements of thin films prepared with raw and ultrasonicated lacquer sap solutions.

As shown in Fig. 8a, the pencil hardness of the raw lacquer film was measured to be H according to ASTM D3363. The pencil hardness of the lacquer films as cast from the lacquer sap solution treated for 15, 30, 45, and 60 min with strengths of 15 W and 100 W are shown as H, H, 2H, and H, and H, 2H, H, and HB, respectively (Fig. 8a, Table 2). The hardness was slightly improved to 2H by ultrasonication-driven prepolymerisation of the lacquer sap solution. It is noted that 17 grades from 6B ~ B-HB-F-H ~ 9H were used to test the film hardness. The smaller W/O emulsion droplets of the lacquer sap solution generated by ultrasonication accelerated urushiol cross-linking in all directions. As a result, the film became denser and the hardness gradually increased [19]. The adhesion level of the lacquer films was determined by the cross-cut method for assessing the resistance of films to separation from substrates (Fig. 8b–d). The samples were prepared by coating the lacquer sap solution on the wood substrate, and their adhesion was measured using a cross cutter and a dedicated tape. The peeling area ratio of the raw lacquer-film was 15.37%. However, the peeling area ratios of the ultrasonicated lacquer-films treated for 15, 30, 45, and 60 min with power intensities of 15 W and 100 W were estimated to be 11.76, 9.99, 9.74, and 10.52 and 8.9, 8.71, 9.49, and 10.22%, respectively (Fig. 8b). The adhesion was increased compared to that of raw lacquer film. Interestingly, the lacquer sap solution composed of smaller emulsion W/O droplets prepared by stronger ultrasonic waves showed stronger adhesion. In general, it is known that the phenol moiety having a hydroxyl group in urushiol influences the adhesion while fixing the lacquer to the surface of the substrate [12], [31]. For the lacquer sap solution treated with ultrasonic waves, the emulsion droplet size decreased significantly and the density of emulsion particles per unit area increased. A relatively large number of phenol groups that can adhere to the substrate surface was generated, resulting in an increase in adhesion. The data are summarised in Table 3. According to ASTM D3359, the best grade is 5B, followed by 4B, 3B, 2B, 1B, and 0B grade.

**Table 3:** Adhesion level of lacquer thin films coated on substrates of red pine wood.

Samples	Ultrasonication (min)	Coating area (mm^2^)	Attached area (mm^2^)	Peeled area (%)	Classification (ASTM D3359)
Lacquer sap solution	0	100	84.63	15.37	2B
15 W treatment	15	100	88.24	11.76	3B
	30	100	90.01	9.99	3B
	45	100	90.26	9.74	3B
	60	100	89.48	10.52	3B
100 W treatment	15	100	91.10	8.90	3B
	30	100	91.29	8.71	3B
	45	100	90.51	9.49	3B
	60	100	89.78	10.22	3B

## 4 Conclusions

The W/O emulsion of lacquer sap was uniformly pulverised by ultrasonication, forming a large number of droplets of submicron sizes. The relatively increased interface between oil and water by nano-emulsification facilitated the polymerisation of urushiol, including enzyme-catalysed oxidation and aerobic oxidation, compared to that of raw lacquer sap, enabling rapid drying of lacquer sap. Increased contact between urushiol and laccase accelerated laccase-catalysed oxidation, suppressing discolouration when drying the lacquer sap. The formation of smaller W/O emulsion droplets leads to improved lacquer film transmittance due to the reduction in light scattering. The increased contact between the emulsion droplets promoted prepolymerisation, resulting in improved film hardness by forming a three-dimensional high-density polymer network of urushiol. The phenyl ring of urushiol, which was considerably exposed to the substrate surface, increased its ability to adhere to the substrates during drying. This method shortens the drying time of lacquer sap and improves the characteristics of lacquer film without any chemical modification, ensuring a low cost, simple process, and broad applicability of the oriental lacquer sap as an eco-friendly natural resin.

## Funding

This work was supported by the Ministry of Culture, Sports, and Tourism (MCST) and Korea Creative Content Agency (KOCCA) in the Culture Technology (CT) Research & Development Program 2019 (R2019020040). This work was supported by a National Research Foundation (NRF) grant funded by the Ministry of Education (2019R1A2B5B01070463) of the Korean government and “Nobel Research Project” grant of the Grubbs Center for Polymers and Catalysis funded by the GIST in 2021. We acknowledge Ms. Hanna Lee for help in preparing the brush-coated lacquer films.

## CRediT authorship contribution statement

**Hyo-Jun Oh:** Investigation, Formal analysis, Data curation. **Jun Ho Hwang:** Investigation, Formal analysis, Software. **Minju Park:** Validation. **Soo Jin Kim:** Investigation, Validation. **Jihoo Lee:** Methodology, Visualization. **Ho Sun Lim:** Methodology. **Sang-Soo Lee:** Funding acquisition. **Jung Ah Lim:** Visualization, Supervision. **Eunji Lee:** Conceptualization, Data curation, Writing - review & editing, Visualization, Supervision, Project administration.

## Declaration of Competing Interest

The authors declare that they have no known competing financial interests or personal relationships that could have appeared to influence the work reported in this paper.
